# Association of Race/Ethnicity With Likeliness of COVID-19 Vaccine Uptake Among Health Workers and the General Population in the San Francisco Bay Area

**DOI:** 10.1001/jamainternmed.2021.1445

**Published:** 2021-03-30

**Authors:** Kevin Grumbach, Timothy Judson, Manisha Desai, Vivek Jain, Christina Lindan, Sarah B. Doernberg, Marisa Holubar

**Affiliations:** 1 Department of Family and Community Medicine, University of California, San Francisco; 2 Division of Hospital Medicine, Department of Medicine, University of California, San Francisco; 3 Quantitative Sciences Unit, Department of Medicine, Stanford University, Stanford, California; 4 Division of HIV, Infectious Diseases & Global Medicine, San Francisco General Hospital, San Francisco, California; 5 Department of Epidemiology and Biostatistics, University of California, San Francisco; 6 Division of Infectious Diseases, Department of Medicine, University of California, San Francisco; 7 Division of Infectious Diseases and Geographic Medicine, Stanford University School of Medicine, Stanford, California

## Abstract

This cross-sectional survey study investigates COVID-19 vaccine intentions among racially and ethnically diverse samples of health workers and the general population.

Surveys have demonstrated racial differences in the public’s willingness to receive a COVID-19 vaccine^1,2^ but have not directly compared vaccine intentions among health workers and the general public.^3^ We investigated COVID-19 vaccine intentions among racially and ethnically diverse samples of health workers and the general population.

## Methods

We conducted a cross-sectional survey from November 27, 2020, to January 15, 2021, nested within 2 longitudinal cohort studies of prevalence and incidence of SARS-CoV-2 infection in 6 San Francisco Bay Area counties. The general population cohort comprised 3935 community-residing adults sampled from randomly selected households, and the medical center employee cohort comprised 2501 employees of 3 large medical centers, who volunteered for biweekly to monthly COVID-19 testing. The main outcome measure was likeliness of vaccine uptake, derived from 2 survey items: (1) “How likely are you to get an approved COVID-19 vaccine when it becomes available?” (using a 1-7 Likert scale anchored at “not at all likely” and “very likely”), and (2) “How early would you ideally like to receive the COVID-19 vaccine?” (asked of respondents scoring ≥3 on the first item). The survey also included items asking about reasons to get, and to not get, vaccinated. Respondents self-identified race/ethnicity (see eMethods in the Supplement for details on sampling and the survey instrument). Crude results were compared using 2-tailed χ^2^ tests, with *P* < .05 considered significant. Logistic regression models stratified by cohort tested association of race/ethnicity with vaccine willingness, adjusting for age, gender, and level of education. All statistical analyses were performed using SAS, version 9.4 (SAS Institute). American Association for Public Opinion Research Response Rate 1 definition was used.

The University of California, San Francisco, and Stanford Institutional Review Boards designated the general population cohort study a public health surveillance study and approved the medical center employee cohort study protocol. Written electronic informed consent was obtained at enrollment.

## Results

A total of 3161 of 3935 (80.3%) participants in the general population cohort and 1803 of 2501 (72.1%) participants in the medical center employee cohort responded to the vaccine survey (Table). Although a higher proportion of medical center employees than members of the general population reported likeliness of vaccine uptake, racial/ethnic differences in likeliness were comparable in both cohorts (Figure). In the medical center cohort, the adjusted odds ratio (aOR) (95% CI) of likeliness of vaccine uptake relative to White respondents was 0.24 (0.10-0.60) for Black respondents, 0.50 (0.31-0.79) for Latinx respondents, 0.37 (0.27-0.51) for Asian respondents, 0.28 (0.15-0.53) for respondents of other races, and 0.49 (0.29-0.82) for respondents of multiple races. In the general population cohort, the aOR (95% CI) relative to White respondents was 0.29 (0.20-0.43) for Black respondents, 0.55 (0.43-0.71) for Latinx respondents, 0.57 (0.47-0.70) for Asian respondents, 0.62 (0.38-1.02) for respondents of other races, and 0.65 (0.46-0.92) for respondents of multiple races. Ratings of reasons to get vaccinated were similar across racial/ethnic groups, but Black, Latinx, and Asian respondents were significantly more likely than White respondents to endorse reasons to not get vaccinated, especially less confidence in the vaccine preventing COVID-19 (aOR [95% CI] for Black, Latinx, and Asian respondents having low confidence relative to White respondents, 2.39 [1.58-3.61], 2.04 [1.58-2.64], and 1.85 [1.51-2.27], respectively); less trust in companies making the vaccine (aOR [95% CI] for Black, Latinx, and Asian respondents having low trust relative to White respondents, 3.08 [2.00-4.73], 1.85 [1.38-2.48], and 1.34 [1.04-1.72], respectively); and more worry that government rushed the approval process (aOR [95% CI] for Black, Latinx, and Asian respondents relative to White respondents, 2.10 [1.44-3.05], 1.68 [1.34-2.10], and 1.81 [1.53-2.15], respectively).

**Table.** Characteristics of Respondents in the Medical Center Employee and General Population Cohorts

Characteristic	No. (%)		*P* value^a^
	Medical center employee cohort (n = 1803)	General population cohort (n = 3161)	
Age categories			
18-39 y	898 (49.8)	885 (28.0)	<.001
40-64 y	851 (47.2)	1534 (48.5)	
≥65 y	45 (2.5)	742 (23.5)	
Unknown	9 (0.5)	0	
Gender			
Female	1348 (74.8)	1702 (53.8)	NA
Male	343 (19.0)	1431 (45.3)	
Other	8 (0.4)	27 (0.9)	
Unknown	104 (5.8)	1 (0)	
Race/ethnicity group			
White	989 (54.9)	1928 (61.0)	<.001
Black	23 (1.3)	116 (3.7)	
Hispanic/Latinx	154 (8.5)	312 (9.9)	
Asian	365 (20.2)	575 (18.2)	
Multiple races	105 (5.8)	154 (4.9)	
Other	50 (2.8)	73 (2.3)	
Unknown	117 (6.5)	3 (0.1)	
Education			
Less than college	18 (1.0)	340 (10.8)	<.001
College	689 (38.2)	1506 (47.6)	
Higher than college	979 (54.3)	1261 (39.9)	
Unknown	117 (6.5)	54 (1.7)	
Occupation			
Physician, advanced practitioner, nurse	1382 (76.7)	NA	NA
Pharmacist, therapist, technician	217 (12.0)	NA	
Other medical center occupation	204 (11.3)	NA	
Employed in health sector	NA	258 (8.2)	
Not employed in health sector	NA	2903 (91.8)	
Likeliness of vaccine uptake	1507 (83.6)	2071 (65.5)	<.001

Abbreviation: NA, not applicable.

^a^ *P* values are from χ^2^ tests.

**Figure.**
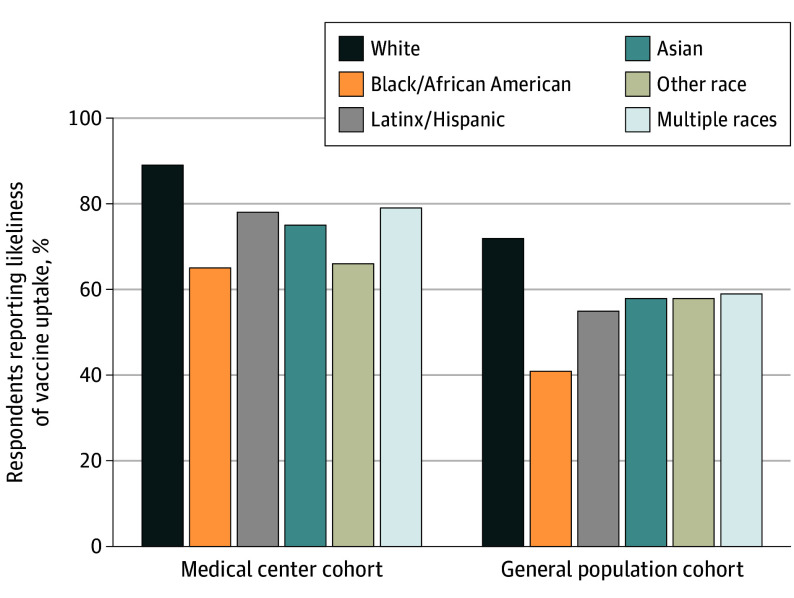
Likeliness of Vaccine Uptake by Cohort and Race/Ethnicity Data shown are crude results.

## Discussion

In this survey study including a diversity of racial/ethnic groups, occupational immersion in a health care setting did not offset disparities in COVID-19 vaccination intentions. We found that Asian individuals, multiracial individuals, and those of other races were more similar to Black and Latinx individuals than White individuals in their likeliness of vaccine uptake. Limitations of this study include that the sample was drawn from people sufficiently concerned about their risk of COVID-19 and trusting of research to volunteer for a study involving repeated COVID-19 testing and the survey not including additional domains, such as perceived access, that might influence reported likeliness of vaccine uptake. However, it is striking that even among individuals motivated to participate in a longitudinal COVID-19 testing study, there were racial/ethnic differences in COVID-19 vaccination intentions and concerns about the vaccine.

Black, Latinx, Asian, and Native American communities have borne a disproportionate toll of the COVID-19 pandemic in the US^4^; inequities in vaccination would compound these disparities. Our survey was fielded at the time of the first emergency use authorization of COVID-19 vaccines in the US. Vaccination rollout since then has revealed barriers to accessing vaccination among historically marginalized populations who are highly motivated to be vaccinated.^5^ Vaccination intentions must be understood as a deliberative and dynamic process; a focus on intentions must not distract from the importance of ensuring equitable access to vaccination.^5^ Special effort is required to reach historically marginalized populations, including those in health occupations, to support informed vaccination decision-making and facilitate access. Efforts must acknowledge a history of racism that has degraded the trustworthiness of health and medical science institutions among historically marginalized populations,^6^ undermined confidence in COVID-19 vaccines, and perpetuated inequitable access to care.

## Notes

Supplement.
eMethods.

eReferences.

## References

[1] Khubchandani J, Sharma S, Price JH, Wiblishauser MJ, Sharma M, Webb FJ. COVID-19 vaccination hesitancy in the United States: a rapid national assessment. J Community Health. 2021;46(2):270-277. doi:10.1007/s10900-020-00958-x 33389421 PMC7778842

[2] Hamel L, Kirzinger A, Muñana C, Brodie M. KFF COVID-19 Vaccine Monitor: December 2020. Accessed March 12, 2021. https://www.kff.org/coronavirus-covid-19/report/kff-covid-19-vaccine-monitor-december-2020/

[3] Shaw J, Stewart T, Anderson KB, . Assessment of US health care personnel (HCP) attitudes towards COVID-19 vaccination in a large university health care system. Clin Infect Dis. Published online January 25, 2021. doi:10.1093/cid/ciab054 33491049 PMC7929026

[4] Rossen LM, Branum AM, Ahmad FB, Sutton P, Anderson RN. Excess deaths associated with COVID-19, by age and race and ethnicity—United States, January 26–October 3, 2020. MMWR Morb Mortal Wkly Rep. 2020;69(42):1522-1527. doi:10.15585/mmwr.mm6942e2 33090978 PMC7583499

[5] Corbie-Smith G. Vaccine hesitancy is a scapegoat for structural racism. JAMA Health Forum. Published online March 25, 2021. doi:10.1001/jamahealthforum.2021.0434 36218456

[6] Cooper LA, Crews DC. COVID-19, racism, and the pursuit of health care and research worthy of trust. J Clin Invest. 2020;130(10):5033-5035. doi:10.1172/JCI141562 32730230 PMC7524489

