# Supplementary material for: Association of Race/Ethnicity With Likeliness of COVID-19 Vaccine Uptake Among Health Workers and the General Population in the San Francisco Bay Area
Source: JAMA Intern Med. 2021 Mar 30;181(7):1008–11. doi: 10.1001/jamainternmed.2021.1445 (PMC8010643; doi:10.1001/jamainternmed.2021.1445)
Supplement: Supplement. — eMethods. eReferences. [file jamainternmed-e211445-s001.pdf]

Supplemental Online Content

Grumbach K, Judson T, Desai M, et al. Association of race/ethnicity with likeliness of COVID-19 vaccine uptake among health workers and the general population in the San Francisco Bay Area. *JAMA Intern Med*. Published online March 30, 2021. doi:10.1001/jamainternmed.2021.1445

eMethods.

eReferences.

This supplemental material has been provided by the authors to give readers additional information about their work.

© 2021 Grumbach K et al. *JAMA Internal Medicine.*

eMethods

Sampling method for cohort enrollment

The General Population Cohort Study used an address-based stratified random sampling strategy to select households in the 6 counties of the San Francisco Bay Area eligible for study recruitment, with enrollment occurring between July and December, 2020. Two strata were considered in the sampling scheme: estimated cases per census tract determined by modeling, and county. Household risk was estimated by modeling prevalent cases within census tracts as reported by counties as a function of sociodemographic, occupational, health and poverty characteristics using data from the 2018 American Community Survey and UCSF Health Atlas.[1] One adult from each randomly selected household was eligible for participation. The Medical Center Employee Cohort study recruited adults employed in diverse occupations by the three medical centers in the San Francisco Bay Area (UCSF Health, Stanford Health Care, and San Francisco General Hospital), enrolled from July through November, 2020.

Survey administration

Participants in both cohorts were sent an electronic survey about COVID-19 vaccination. Surveys were provided in English, Spanish and Chinese languages. Those who did not respond were invited to complete the survey in person at a regular study testing visit. The survey was fielded with the General Population Cohort Study from December 14, 2020 to January 15, 2021, and with the Medical Center Employee Cohort Study from November 27 to December 27, 2020, around the time of the announcements of emergency use authorizations for the Pfizer (December 11, 2020 and Moderna (December 18, 2020) vaccines.

Survey instrument

Vaccination survey instruments were adapted from the NIH Community Engagement Alliance (CEAL) Against COVID-19 Disparities Draft Common Survey,[2] and informed by well-established conceptual models of vaccine hesitancy.[3, 4] A binary measure of likeliness of vaccine uptake was derived from two survey items. The first item asked, "How likely are you to get an approved COVID-19 vaccine when it becomes available?", using a 1-7 Likert scale with 1 indicating "not at all likely" and 7 "very likely." Respondents who scored 3 or greater were asked a second question, "How early would you ideally like to receive the COVID-19 vaccine?", with response options of "I'd like to be among the earliest," "I'd like to receive it early, but not in the first round of people," " I'd like to receive it later in the distribution process," "or "I'd like to wait at least two months to see what the experience is." Respondents who selected 3 or greater on the first item and answered "I'd like to be among the earliest" or "I'd like to receive it early…" to the second item were categorized as having likeliness of vaccine uptake.

Participants received a baseline survey at initial cohort enrollment ascertaining demographic characteristics. Participants self-identified their race-ethnicity using Office of Management and Budget categories, with one question on Hispanic/Latino ethnicity and one on racial identity. Participants could select more than one race. The ethnicity and race items were then combined to create a single race-ethnicity variable with mutually exclusive categories, including a multi-racial category. The few respondents who selected Hispanic/Latino ethnicity and Black race were categorized as Black. We use the term people of color to refer to respondents identifying as Black, Latinx/Hispanic, Asian, multi-racial, and race other than white. Other sociodemographic variables included age, gender, occupation, and highest level of education attained.

© 2021 Grumbach K et al. *JAMA Internal Medicine.*

eReferences

1. UCSF School of Medicine Dean's Office of Population Health and Health Equity. UCSF Health Atlas. Accessed February 16, 2021. https://healthatlas.ucsf.edu/?active=covid_new_cases_percap

2. National Institutes of Health. *Community Engagement Alliance*.; 2020. Accessed February 16, 2021. https://covid19community.nih.gov/about

3. MacDonald NE. Vaccine hesitancy: Definition, scope and determinants. *Vaccine*. 2015;33(34):4161-4164. doi:10.1016/j.vaccine.2015.04.036

4. Larson HJ, Jarrett C, Eckersberger E, Smith DMD, Paterson P. Understanding vaccine hesitancy around vaccines and vaccination from a global perspective: A systematic review of published literature, 2007–2012. *Vaccine*. 2014;32(19):2150-2159. doi:10.1016/j.vaccine.2014.01.081

© 2021 Grumbach K et al. *JAMA Internal Medicine.*
